# Novel Flexible PVDF-TrFE and PVDF-TrFE/ZnO Pressure Sensor: Fabrication, Characterization and Investigation

**DOI:** 10.3390/mi12060602

**Published:** 2021-05-23

**Authors:** Mingran Liu, Yang Liu, Limin Zhou

**Affiliations:** 1 Department of Mechanical Engineering, The Hong Kong Polytechnic University, Hung Hom, Kowloon, Hong Kong, China; mmyliu@polyu.edu.hk; 2 School of System Design and Intelligent Manufacturing, Southern University of Science and Technology, Xueyuan Road 1088, Shenzhen 518055, China; zhoulm@sustech.edu.cn

**Keywords:** PVDF-TrFE, PVDF-TrFE/ZnO, β phase crystal, human pulsation sensor

## Abstract

With the development of human healthcare devices, smart sensors, e-skins, and pressure sensors with outstanding sensitivity, flexibility, durability and biocompatibility have attracted more and more attention. In this paper, to develop a novel flexible pressure sensor with high sensitivity, different poly (vinylidene fluoride-trifluoroethylene) (PVDF-TrFE)-based composite membranes were fabricated, characterized and tested. To improve the β-phase crystallinity and piezoelectricity of the membranes, and for the purpose of comparison, nano ZnO particles with different concentrations (99:1, 9:1 in a weight ratio of PVDF-TrFE to ZnO) were, respectively added into PVDF-TrFE polymer acting as a nucleating agent and dielectric material. To facilitate the formation of β-phase crystal, the membranes were fabricated by electrospinning method. After the electrospinning, an annealing process was conducted to the fabricated membranes to increase the size and content of β-phase crystal. Then, the fabricated PVDF-TrFE membranes, acting as the core sensing layer, were, respectively built into multiple prototype sensors in a sandwich structure. The sensitivity of the prototype sensors was tested by an auto-clicker. The stimulation of the auto-clicker on the prototype sensors generated electrical signals, and the electrical signals were collected by a self-built testing platform powered by LabVIEW. As a result, combining the addition of ZnO nanofillers and the annealing process, a highly sensitive pressure sensor was fabricated. The optimal peak-to-peak voltage response generated from the prototype sensor was 1.788 V which shows a 75% increase compared to that of the pristine PVDF-TrFE sensor. Furthermore, a human pulse waveform was captured by a prototype sensor which exhibits tremendous prospects for application in healthcare devices.

## 1. Introduction

**Figure 1:**
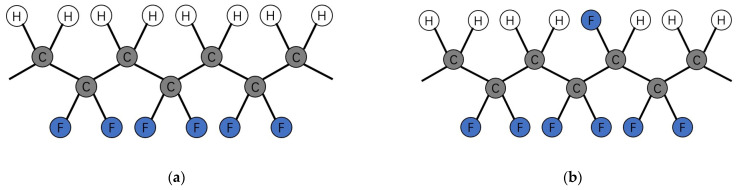
(**a**) Molecular conformation and crystal structures β-phases of PVDF [7]; (**b**) Schematic drawing of VDF-TrFE copolymer molecule in β phase (all-trans) conformation (right image) [8].

In recent years, the development directions of pressure sensors have been subdivided based on physical application scenarios [1]. With the progress of many new emerging areas like wearable devices, e-skins and robots, miniature, flexible and sensitive pressure sensors have become the mainstream direction of research [2,3,4]. In this field, composites based on piezoelectric materials attracted much attention due to their brilliant flexibility, controllable response and tunable mechanical, electrical and chemical properties [4]. In 2015, James S. Lee and his teammates demonstrated a flexible tactile sensor that utilizes a PVDF/ZnO nanorod composite as the sensing material [5]. With their piezoelectric sensor, a change in pressure as small as 10 Pa can be detected. In Lee’s research, PVDF acted as a matrix material in the piezoelectric composite due to its brilliant flexibility and piezoelectricity. Generally, the sensitivity of PVDF-based nanocomposite sensors is dominantly determined by their permittivity and piezoelectricity, which are resultant parameters of the content and size of the β-phase crystal in the fabricated sensing material [6]. Therefore, increasing the content and size of the β-phase crystal shown in Figure 1 is one of the most effective methods to improve the sensitivity of sensing materials. Due to a substitution of a hydrogen atom by a fluorine atom in the molecular chain shown in Figure 1 which facilitates the formation of β-phase crystal, PVDF-TrFE, which is a co-polymer of PVDF with a similar chain structure, could show even stronger piezoelectricity than that of PVDF under the same situation.

Recently in 2020, Li et al., presented poly (vinylidenefluoride-co-trifluoroethylene)/carbon-based nanomaterial composite films for pressure sensing applications [9]. In their study, the PVDF-TrFE-based composite piezoelectric film was treated by polarization and annealing for an improvement of crystallinity and piezoelectric effect. Their study showed an increase in the β-phase content from 73.6% to 86.4% and an increase in the piezoelectric coefficient from 19.8 ± 1.0 to 26.4 ± 1.3 pC/N. However, there is still room for improvement in the fabrication method of the films in their research. In terms of β-phase crystal formation and piezoelectricity of the composite, a fabrication method is as important as material selection. To elongate a composite solution to a membrane with a designated thickness which could keep sufficient flexibility and durability, dip coating and spin coating are the most popular fabrication methods [10]. As is known to all, treatment of stretching and poling performed simultaneously to the PVDF or PVDF-TrFE polymer could significantly induce the formation of β phase crystal to improve its piezoelectricity and dielectricity [11]. Therefore, electrospinning, a membrane fabrication method that could stretch the composite fiber in a strong electric field, would be an attractive method in the fabrication of PVDF-TrFE-based piezoelectric material.

Although previous works of other researchers have achieved satisfactory results, there is still room for improvement in the sensitivity of piezoelectric composite pressure sensors. Therefore, in this study, four measures are adopted to improve sensitivity. Firstly, PVDF-TrFE is used to replace the traditional PVDF to act as the matrix material of the composite to facilitate the formation of β-phase crystal. Secondly, ZnO with its promising dielectric property, biocompatibility, and a substantial effect in the facilitation of crystallization in PVDF-TrFE, is used as the filler material in our novel flexible pressure sensing composite [12]. Thirdly, electrospinning is conducted to fabricate the piezoelectric membrane to maximize the size and content of β-phase crystal. Fourthly, annealing post-treatment is adopted to improve the piezoelectric property of the membrane [13].

Combining all of the abovementioned outcomes, novel flexible pressure sensors made of PVDF-TrFE, PVDF/TrFE/ZnO (99:1, 90:10 in weight ratio of PVDF-TrFE to ZnO) were fabricated by electrospinning in this paper. The morphology of the fabricated samples was characterized by SEM. Phase identification and crystallinity determination of the piezoelectric polymer were conducted by XRD. The performance of the fabricated sensors was tested by a self-built testing platform powered by LabVIEW. As a result, a 1.788 V peak-to-peak voltage was generated by the optimal sample under the auto-clicking test. This voltage value shows a 75% increase than that of the pristine PVDF-TrFE sample. Furthermore, a human pulse waveform was captured by a prototype sensor which exhibits tremendous prospects for application in healthcare devices.

## 2. Materials and Methods

### 2.1. Materials

PVDF-TrFE with the brand name PIEZOTECH^®^FC30 (typical TrFE content (in mol %) is 30) was purchased from Piezotech, Pierre-Bénite, France. Zinc Oxide particles with a diameter of 30 nm were purchased from Aladdin, Shanghai, China. Acetone in ACS Grade was purchased from AQA, Anaqua Global International Inc. Limited, Hong Kong, China. N,N dimethylformamide (DMF) came from International Laboratory USA.

### 2.2. Fabrication

#### 2.2.1. Fabrication of Sensing Materials

To fabricate a sensitive and optimal sensing layer based on PVDF-TrFE and nano-ZnO, eight different comparison groups were designed, fabricated and analyzed in this project. They are annealed and unannealed PVDF-TrFE, annealed and unannealed PVDF-TrFE doped with ZnO particles in different weight ratios, respectively. Abbreviations for eight comparison groups are listed in Table 1.

To completely dissolve the PVDF-TrFE and ZnO particles and control the rate of evaporation during electrospinning, the organic solvent was mixed by DMF and Acetone at a volume ratio of 3:2. Regarding the viscosity of the solution and the quality of the electrospinning product, a 15% weight to volume ratio of the PVDF-TrFE powder to organic solvent was determined after adjustment. Once the organic solvent was completely mixed, PVDF-TrFE powder in 15% *w*/*v* was added to it. For a complete dissolution, the solution was treated by ultrasonic stirring for 1 h and magnetic stirring for 12 h, respectively.

In the preparation of the solution of PVDF-TrFE doped with ZnO in different concentrations, the weight to volume ratio of solute to solvent was kept as 15%, the same as the abovementioned pristine PVDF-TrFE solution. In these PVDF-TrFE/ZnO solutions, the ZnO particles were added into the solvent first. The ZnO solution was treated by ultrasonic stirring for 1 h. Then, the PVDF-TrFE powder was poured into the ZnO solution gradually for full integration. The solutions were treated by ultrasonic stirring for 1 h and magnetic stirring for 12 h, respectively, the same as the previously mentioned procedure.

In this project, a 20 mL prepared solution with four different compositions was poured into a syringe for further electrospinning. Several key setting parameters that can dramatically influence the process of electrospinning should be considered based on the material used and the environment of the laboratory such as temperature and humidity [14]. Therefore, after multiple trials and analyses, the setting of the electrospinning was determined. The voltage between the needle of the syringe and the collector which can create the high electric field was set as 20 kV. The feed rate of the solution in the syringe which could ensure a uniform and continuous nanofiber was set as 0.036 mL/min. The distance between the needle of the syringe and the electrospinning fiber collector which determines the distance of ejected solution flight and volatilization time was 15 cm. The relative humidity must be kept between 20% and 50%. After the electrospinning, an annealing process for the electrospinning membranes was conducted at 120 °C for one hour.

#### 2.2.2. Fabrication of Prototype Sensors

**Figure 2:**
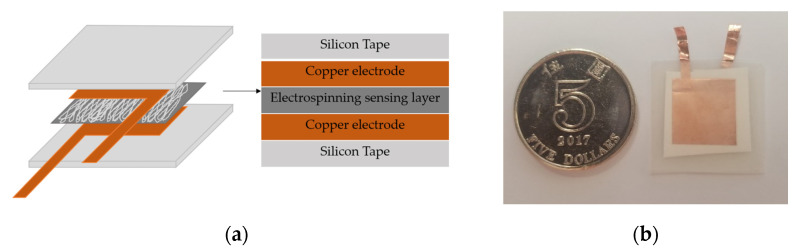
(**a**) Diagram and (**b**) image of the prototype sensor compared with a coin.

In this project, a simple stacking sandwich structure was applied to test the piezoelectric response of the electrospinning membranes. The upper and lower parts of the sensor were composed of ultrathin flexible copper electrodes with the size of 15 mm ×15 mm. The middle part of the sensor was the sensing layer with the size of 18 mm × 18 mm fabricated by electrospinning. As shown in Figure 2, the stacked sensor was encapsulated by the flexible silicon rubber tape with the size of 25 mm ×25 mm.

### 2.3. Characterization

A Scanning Electron Microscope (Tescan VEGA3) was used for the morphology analysis of the nanofiber fabricated by electrospinning. A X-Ray Diffractometer (Rigaku SmartLab 9) was applied for the phase identification and crystallinity determination of the electrospinning fibers.

### 2.4. Piezoelectric Response Test Method

The piezoelectric response of the fabricated sensors was validated by a continuous clicking test. The stimuli from an auto-clicking device with a frequency of 2 hz were allowed to continuously hit the tested sensors. The electrical signal generated by hitting was collected by a data acquisition device and transferred into a computer. A virtual oscilloscope based on LabVIEW was built up and applied for recording and processing the information of the collected signal. In addition to the continuous clicking test, a pulse capture test was conducted for the sensor consisting of a PT10Zad sensing layer which has produced optimal piezoelectric response during the previous tests. By utilizing the PT10Zad sample which has the optimal sensitivity to stimulus, the human pulse pressure with a relatively weak pressure signal was captured. The sensor was mounted onto the wrist of human body which mimics the manner of palpation. As this is a prototype sensor, assistance by hand is necessary for positioning the pulse. Then, human pulse signals can be captured by the sensor and transferred to the computer for further processing.

## 3. Results and Analysis

### 3.1. SEM Results

**Figure 3:**
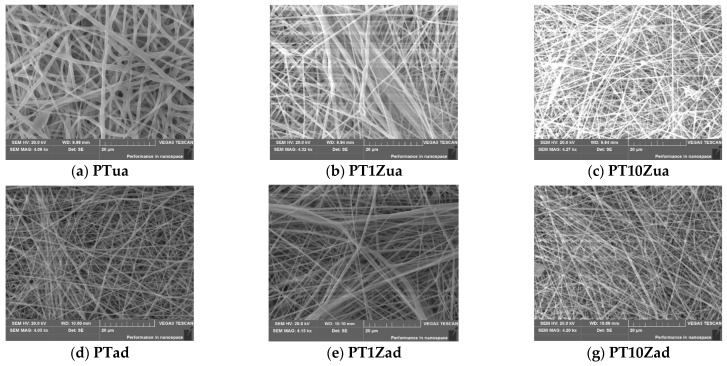
(**a**–**g**) SEM figures of the unannealed and annealed electrospinning membrane samples made of PVDF-TrFE doped with 0/1/10 wt% ZnO nanoparticles

The SEM results for all eight samples are shown in Figure 3.

**Figure 4:**
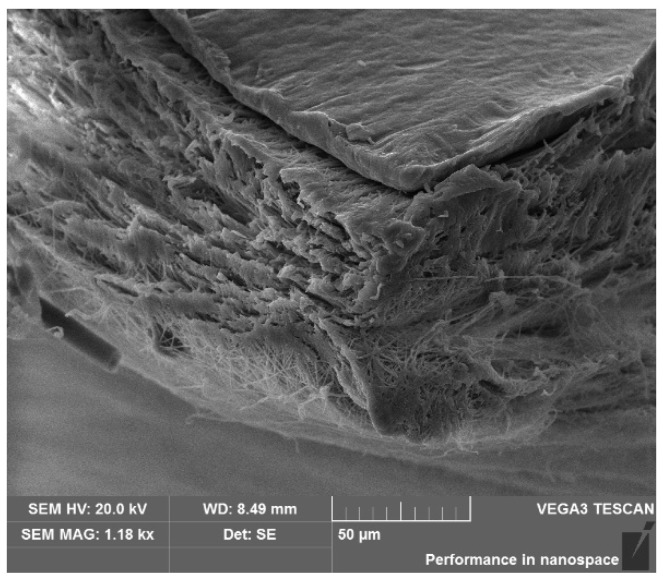
The sectional view of sample PT10Zad.

By analyzing the SEM images for all samples, we can conclude that:

1. Morphology of the electrospinning membranes is clearly captured by the SEM. The flexible membranes composed of fiber structure were well fabricated by electrospinning. The average diameter of the electrospinning fiber is 200~300 nm. The thickness of the membrane is 40~50 μm as shown in Figure 4;
2. The degree of alignment of annealed samples is notably higher than that of the unannealed samples. The bend and knots of the fibers decreased by the treatment of annealing. The improvement of the degree of alignment would induce a significant increase in the piezoelectric performance of the fabricated sensors;
3. With the increase in the weight ratio of nano ZnO particles in different samples, bead structure which was validated as Zinc and oxygen by EDS analysis occurred and its amount increased. These ZnO structures would improve the piezoelectricity of the fabricated membrane by acting as not only a dielectric material but also a nucleating agent to facilitate the formation of β phase crystal in the polymer [12].

### 3.2. XRD Results

**Figure 5:**
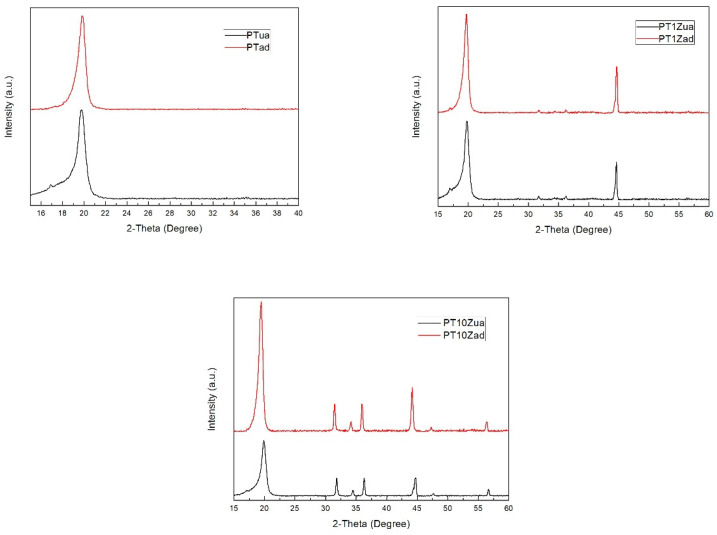
XRD diagrams for eight comparison groups.

By analyzing the XRD diagrams, the elemental composition of the electrospinning membrane can be obtained. Additionally, the size of β phase crystal which dominantly determines the piezoelectricity of PVDF-TrFE-based membrane can be calculated based on the XRD diagrams [6]. The XRD diagrams are shown in Figure 5.

In these diagrams, an obvious increase in the magnitude of the diffraction peaks can be observed at both the 18.2° (202) corresponding to the α-phase and 20.8° (110/220) corresponding to the β-phase PVDF crystal with the annealing treatment and the addition of nano ZnO fillers. The peaks on 36.16°, 47.58°, and 56.62° for all PT/Z samples corresponding to (101), (102), and (110) planes confirmed the formation of a ZnO nanostructure. The increase in the magnitude of ZnO diffraction corresponds to the increase in the concentration of ZnO nanofillers.

Based on the XRD diagram, the full width at half maxima (FWHM) can be measured. Then, by substituting the FWHM and the theta degree of corresponding diffraction peak into the Scherrer equation shown below, the crystal size can be calculated.

(1) $$\tau =\frac{K\lambda }{\beta \cos\theta }$$

where: *K* is a dimensionless shape factor, with a value of 0.89; 𝜆 is the X-ray wavelength which equals 0.15046 nm; *β* is the full width at half maxima (FWHM) and 𝜃 is the Bragg angle.

**Figure 6:**
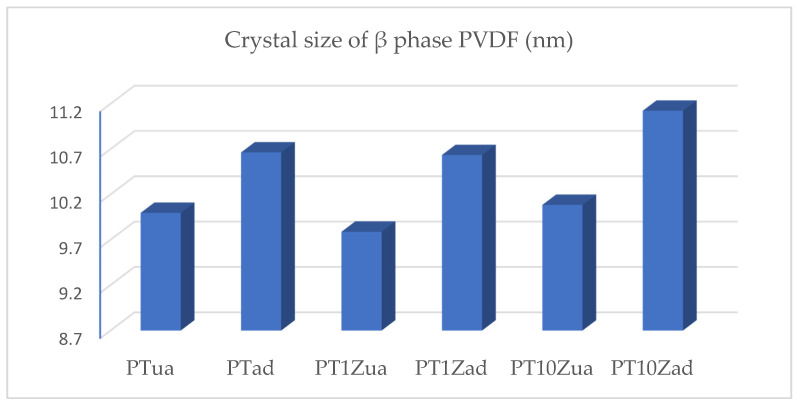
Histogram for β phase crystal size.

Table 2 and Figure 6 listed below present the calculation results for all eight samples.

By analyzing the XRD calculation results, a limited nucleating agent effect of the nano ZnO in PVDF-TrFE polymer can be observed. Compared with the nucleating agent effect of ZnO, the annealing treatment has induced a much stronger coagulation effect of the β phase PVDF crystal.

### 3.3. Piezoelectric Response Test Results

**Figure 7:**
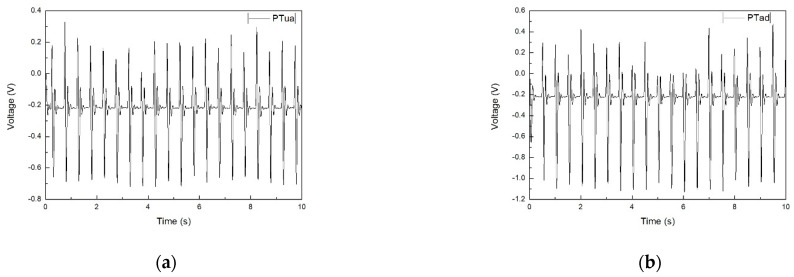

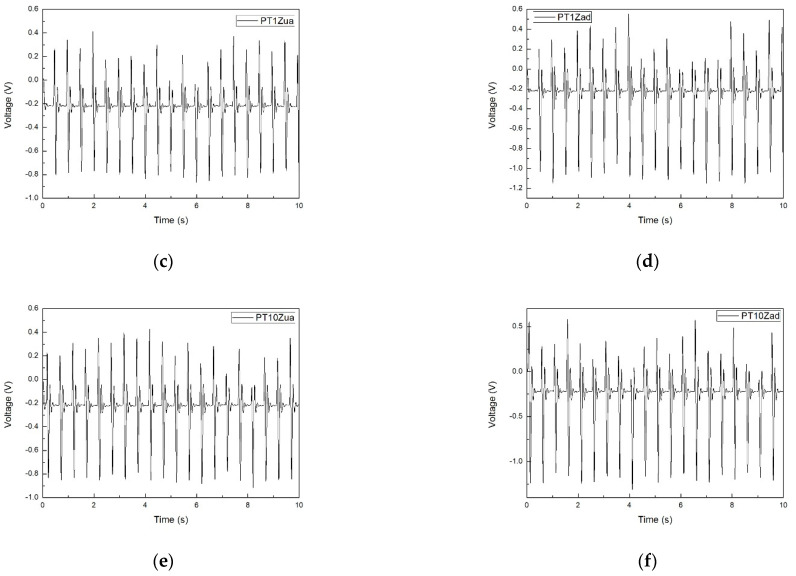
(**a**–**f**) The auto-clicking test results for six samples.

The auto-clicking test on the prototype sensors is conducted for measurement of their sensitivity to constant clicking. By comparing the test results, an optimal composition of the sensing material can be obtained. The results of the auto-clicking test are shown in Figure 7.

**Figure 8:**
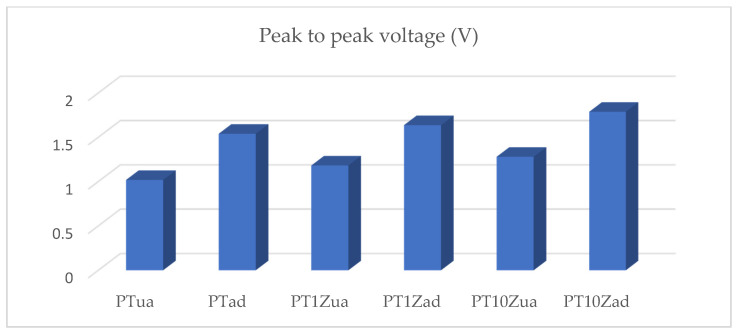
Histogram for peak-to-peak voltage.

For facilitating a comparison among the performance of all six samples, the peak-to-peak voltage for all samples was measured and is listed in Table 3 and Figure 8.

In the auto-clicking test, the recovery time of all samples, which varied between 300 and 397 ms was measured. The addition of ZnO nanofillers induced an increase in peak-to-peak voltage response due to the nucleating agent effect and inherent high permittivity of ZnO. The annealing process showed a significant increase in the sample membranes’ piezoelectric response. This increase was mainly caused by the coagulation effect which was validated by the XRD results and the alignment effect which was validated by the SEM images. Combining the addition of nano ZnO and the annealing process, the optimal peak-to-peak voltage response from sample PT10Zad was 1.788 V which, shows a 75% improvement compared to that of the pristine PT.

**Figure 9:**
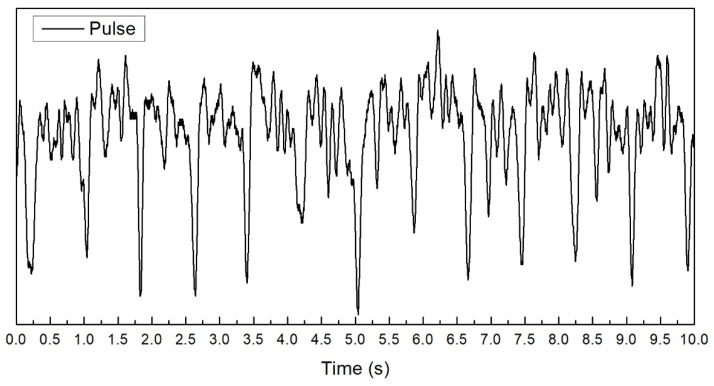
The human pulse waveform captured by sample PT10Zad.

In addition to the auto-clicking tests, a human pulse test was also conducted for the PT10Zad sample which has the best performance in the auto-clicking test. The captured signal of a human pulse capturing is shown in Figure 9. The human pulse with a frequency of around 1 Hz can be captured and identified easily.

## 4. Conclusions

In this paper, novel piezoelectric PVDF-TrFE and PVDF-TrFE-ZnO membranes were fabricated by electrospinning, characterized and investigated. To test the piezoelectric response of the fabricated membranes, the pressure sensors in a simple sandwich structure were designed and fabricated. From the characterization of SEM and XRD and piezoelectric response test results, the conclusions of this research can be drawn. The PVDF-TrFE and PVDF-TrFE-doped ZnO with two different weight ratios (99:1, 90:10) were well fabricated into membranes by electrospinning. The fibers in the membranes were fully elongated and polarized so that the β phase crystal can be transformed and stabilized as much as possible. The annealing treatment for the electrospinning membranes significantly improved their piezoelectric response in two respects: 1. improving the degree of alignment of the fibers in membranes by reducing their bend and knots and 2. increasing the size of the β phase crystal which was validated by XRD results. The addition of the ZnO nanofillers played two roles which were nucleating agent to enlarge the size of the β phase crystal and dielectric material to increase the piezoelectric response. Through the auto-clicking test for all eight samples, the efforts of ZnO nanofillers and annealing treatment emphasized above were validated again. Combining the addition of nano ZnO and the annealing process, the optimal peak-to-peak voltage response from sample PT10Zad was 1.788 V which shows a 75% improvement compared to that of the pristine PT. At last, a clear human pulse waveform was captured by the sample PT10Zad.

These conclusions and results provide a path to the development of flexible pressure sensors. In this paper, the feasibility and potential of the novel electrospinning sensing membranes were confirmed. In the future, the industries of humanoid robots, e-skins, vital sign monitoring, etc. would be the potential application scenarios of this investigation.

## Figures and Tables

**Table 1:** Abbreviations for eight comparison groups in the research.

-	PVDF-TrFE (PT)	PVDF-TrFE to ZnO (99:1)	PVDF-TrFE to ZnO (90:10)
unannealed	PTua	PT1Zua	PT10Zua
annealed	PTad	PT1Zad	PT10Zad

**Table 2:** β phase crystal size calculation results.

Sample	K	λ (nm)	β (Rad)	θ (Deg)	τ (nm)
PTua	0.89	0.154056	0.799	10.4	9.996
PTad	0.89	0.154056	0.749	10.4	10.664
PT1Zua	0.89	0.154056	0.816	10.4	9.788
PT1Zad	0.89	0.154056	0.751	10.4	10.635
PT10Zua	0.89	0.154056	0.792	10.4	10.085
PT10Zad	0.89	0.154056	0.718	10.4	11.124

**Table 3:** Peak-to-peak voltage table.

	Peak to Peak Voltage (V)	Recovery Time (ms)
PTua	1.019	300
PTad	1.54	397
PT1Zua	1.182	332
PT1Zad	1.637	307
PT10Zua	1.28	350
PT10Zad	1.788	355
